# Machine learning for the diagnosis accuracy of bipolar disorder: a systematic review and meta-analysis

**DOI:** 10.3389/fpsyt.2024.1515549

**Published:** 2025-01-28

**Authors:** Yi Pan, Pushi Wang, Bowen Xue, Yanbin Liu, Xinhua Shen, Shiliang Wang, Xing Wang

**Affiliations:** ^1^ Department of Neurosis and Psychosomatic Diseases, Huzhou Third Municipal Hospital, The Affiliated Hospital of Huzhou University, Huzhou, Zhejiang, China; ^2^ Department of Mental Disorders, National Center for Mental Health, NCMHC, Beijing, China; ^3^ Affiliated Mental Health Center & Hangzhou Seventh People’s Hospital, Zhejiang University School of Medicine, Hangzhou, Zhejiang, China

**Keywords:** depression, bipolar disorder, machine learning, predictive model, systematic review

## Abstract

**Background:**

Diagnosing bipolar disorder poses a challenge in clinical practice and demands a substantial time investment. With the growing utilization of artificial intelligence in mental health, researchers are endeavoring to create AI-based diagnostic models. In this context, some researchers have sought to develop machine learning models for bipolar disorder diagnosis. Nevertheless, the accuracy of these diagnoses remains a subject of controversy. Consequently, we conducted this systematic review to comprehensively assess the diagnostic value of machine learning in the context of bipolar disorder.

**Methods:**

We searched PubMed, Embase, Cochrane, and Web of Science, with the search ending on April 1, 2023. QUADAS-2 was applied to assess the quality of the literature included. In addition, we employed a bivariate mixed-effects model for the meta-analysis.

**Results:**

18 studies were included, covering 3152 participants, including 1858 cases of bipolar disorder. 28 machine learning models were encompassed. Sensitivity and specificity in discriminating between bipolar disorder and normal individuals were 0.88 (9.5% CI: 0.74~0.95) and 0.89 (95% CI: 0.73~0.96) respectively, and the SROC curve was 0.94(95% CI: 0.92~0.96). The sensitivity and specificity for distinguishing between bipolar disorder and depression were 0.84 (95%CI: 0.80~0.87) and 0.82 (95%CI: 0.75~0.88) respectively. The SROC curve was 0.89 (95%CI: 0.86~0.91).

**Conclusions:**

Machine learning methods can be employed for discriminating and diagnosing bipolar disorder. However, in current research, they are predominantly utilized for binary classification tasks, limiting their progress in clinical practice. Therefore, in future studies, we anticipate the development of more multi-class classification tasks to enhance the clinical applicability of these methods.

**Systematic review registration:**

https://www.crd.york.ac.uk/prospero/display_record.php?ID=CRD42023427290, identifier CRD42023427290.

## Introduction

Bipolar disorder (BD) is a prevalent, chronic, and frequently recurring mood disorder characterized by intermittent or cycling episodes of mania/hypomania and depression. The prevalence rate is approximately 1%-1.5%, with bipolar disorder often manifesting early, commonly during adolescence. This disorder significantly impacts psychosocial functioning and may result in a potential loss of life expectancy of about 10-20 years (1). Additionally, bipolar disorder is a leading cause of disability in young individuals due to its association with cognitive and functional impairments, and an increased risk of suicide (2).

The symptoms of unipolar disorder and bipolar disorder are similar during depressive episodes, and clinical diagnosis typically relies on collecting medical history and conducting a mental examination. Differentiating between the two can be quite challenging, and some studies indicate an average latency period of 5-10 years from onset to accurate diagnosis and treatment (3).

Nearly 60% of bipolar disorder cases are initially diagnosed as depressive disorder. A study by LEAO I A and others revealed that patients typically consult an average of 4 doctors before receiving a correct bipolar disorder diagnosis (4). In the subsequent treatment process, the treatment regimens also vary. Studies have found that only 20% of patients with bipolar disorder receive appropriate treatment during their initial depressive episode (4). Misdiagnosing bipolar disorder as depression may result in inadequate treatment, a poorer prognosis, higher medical costs, and serious adverse events such as switching to manic episodes or increased suicide tendencies (5), underscoring the importance of early identification of bipolar disorder.

Currently, no single indicator definitively diagnoses bipolar disorder, necessitating a comprehensive analysis that combines clinical experience with other biochemical indicators. Some studies (6–10) suggest inconsistencies in psychological assessments, blood indicators, and neuroimaging between bipolar disorder, depression, and healthy populations. Some researchers (11) posit that patients with bipolar disorder exhibit abnormally reduced activity in the ventrolateral prefrontal cortex during emotional processing and increased activity in the amygdala, striatum, and medial prefrontal cortex. To effectively diagnose bipolar disorder, a variety of methods are needed, such as the use of data from psychological assessments, blood indicators, and neuroimaging.

In recent years, owing to the rapid advancement of computers and the ongoing refinement of statistical theory, artificial intelligence methods have progressively found applications in clinical practice. Numerous researchers are endeavoring to devise discriminative diagnostic methods for bipolar disorder, leveraging the efficiency of machine learning techniques in disease diagnosis and prognosis. However, these attempts often lack adequate evidence-based support. The objective of this study is to conduct a systematic review and meta-analysis to comprehensively assess the potential of various methods in predicting bipolar disorder, evaluate the likelihood of accurate diagnosis, and contemplate the feasibility of integrating multiple methods.

## Materials and methods

### Study registration

This systematic review was conducted in accordance with the reporting guidelines for systematic reviews and meta-analyses (PRISMA 2020) (12), and prospectively registered with PROSPERO (ID: CRD42023427290).

#### Eligibility criteria

Inclusion criteria: (1) Study population: individuals with postpartum depression, aged >18 years; (2) Study content: predictive models constructed for bipolar disorder based on different machine learning methods, with study types including cohort studies, case-control studies, and cross-section studies; (3) English publication.

#### Exclusion criteria

Exclusion criteria: (1) Duplicate publication; (2) Unavailability of full text; (3) Outlines, comments, conference papers, and abstracts; (4) Case reports; (5) Animal experiments.

### Data sources and search strategy

We systematically conducted searches in PubMed, Embase, Cochrane, and Web of Science using relevant subject terms and free-text terms, without geographical or temporal restrictions. The search encompasses the period from the establishment of the database to April 1, 2023. Detailed search materials are included in the **Supplementary Materials**.

### Study selection

The retrieved articles were imported into EndNote, and both software and manual methods were employed to identify and eliminate duplicate original publications. Subsequently, the primary studies meeting the criteria were preliminarily selected based on titles and abstracts. Full texts were downloaded for thorough examination, and the final inclusion of literature was determined while organizing the data. Throughout the literature screening process, two researchers (Wang Xing, a clinician with 10 years of work experience, and Pan Yi, a pharmacist with 10 years of research experience) independently cross-checked the documents. Any disputes were resolved with the assistance of a third party (Shen Zhongxia, a clinician with 20 years of work experience).

### Data extraction

Before initiating the data extraction, we formulated a standardized data extraction form. The extracted content encompasses the first author, publication year, author’s country, study type, subject source, bipolar disorder diagnostic criteria, depression diagnostic criteria, number of samples from the normal population, depression sample number, number of bipolar disorder cases, total number of subjects, number of cases from the normal population in the training set, the number of cases of depression in the training set, the number of cases of bipolar disorder in the training set, the total number of cases in the training set, validation set generation method, the number of cases of unipolar disorder in the validation set, the number of normal cases in the validation set, the number of cases of bipolar disorder in the validation set, total number of subjects in the validation set, type of model used, modeling variables, information on whether compared with clinicians, and modeling variables.

The data extraction process was independently carried out by two researchers (Pan Yi, a clinician with 10 years of work experience, and Xue Bowen, a clinician with 1 years of research experience), and cross-checked after extraction. In the event of any disputes, a third researcher (liu yanbin, a clinician with 11 years of work experience) was invited to assist in the resolution.

### Risk of bias in studies

Following the QUADAS-2 criteria (13), each included study underwent a detailed evaluation, with items categorized as “yes,” “no,” or “unclear.” “Yes” signified compliance with the item, “no” indicated non-compliance or omission, and instances of partial compliance or insufficient information from the literature were assessed as “unclear.” The entire quality assessment process was independently conducted by two researchers (Wang Xing, a clinician with 10 years of experience, and Pan Yi, a pharmacist with 5 years of research experience). In case of disagreements, a third researcher (liu yanbin, a clinician with 11 years of experience) was consulted to assist in reaching a final decision.

### Synthesis methods

Initially, heterogeneity within the included studies was assessed, considering both threshold effects and non-threshold effects. The presence of threshold effects was identified through the construction of a summary receiver operating characteristic (SROC) curve. A “shoulder-arm” distribution on the curve indicated the existence of a threshold effect, while its absence was indicated by a different pattern. The size of heterogeneity was assessed using the Chi-square test or Cochrane-Q test. Based on the results of the heterogeneity test, an appropriate effect model was selected to calculate the combined effect size of the included studies, including sensitivity (correctly predicted bipolar disorder patients/actual bipolar disorder patients), specificity (predicted non-bipolar disorder patients/actual non-bipolar disorder patients), positive likelihood ratio, negative likelihood ratio, and diagnostic odds ratio (the ratio of positive likelihood in identified bipolar disorder patients to the ratio of positive likelihood in non-bipolar disorder patients). Subsequently, the SROC curve was drawn, and the area under the SROC curve (AUC) was calculated to evaluate the overall diagnostic accuracy in predicting bipolar disorder patients. All statistical analyses were conducted using Stata software.

## Results

### Study selection

**Figure 1:**
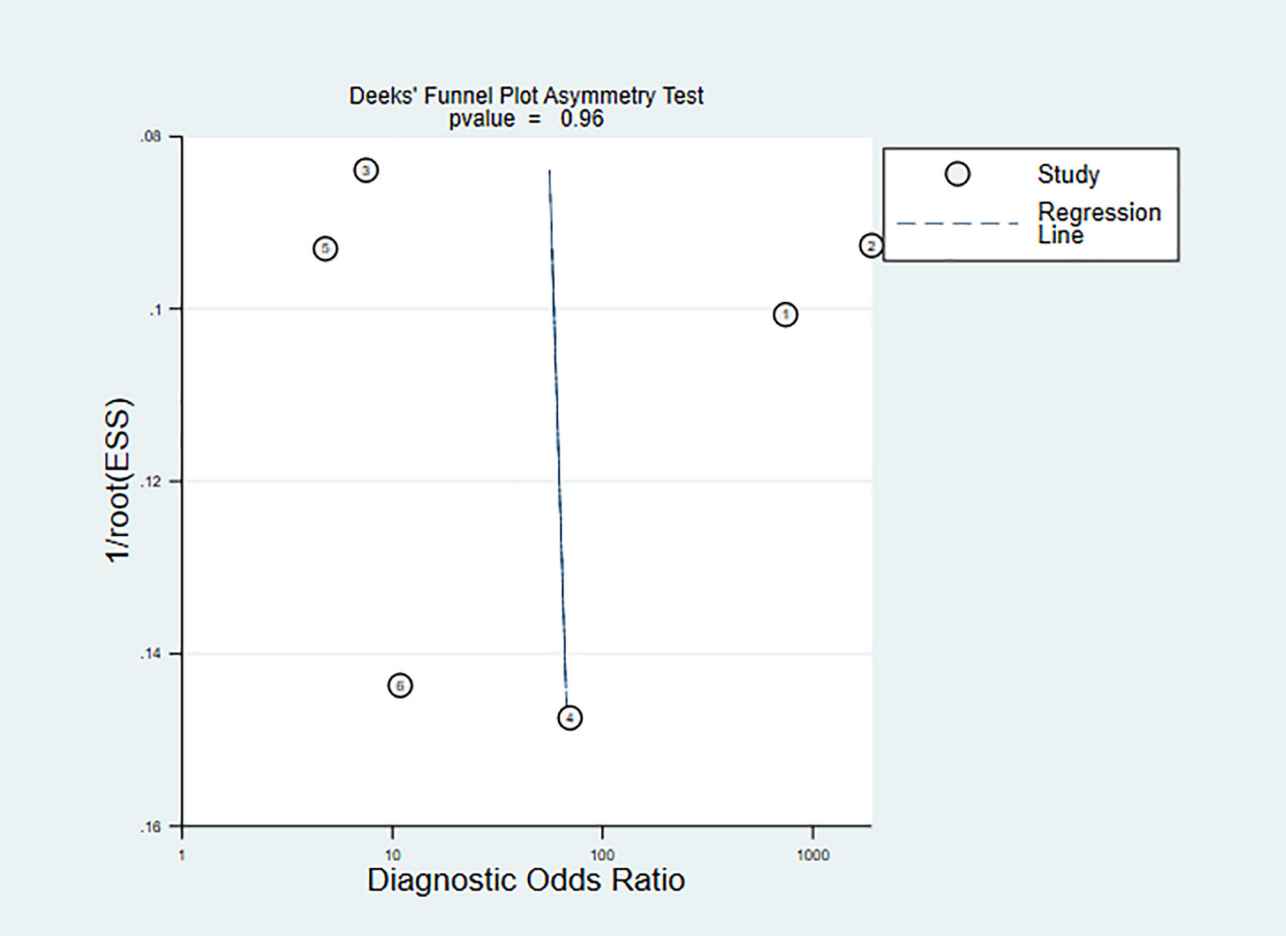
Literature screening process.

A total of 3893 literature pieces were initially retrieved from the database, with 806 duplicates removed during deduplication. Subsequent review of titles and abstracts resulted in the exclusion of 3048 articles. Among the remaining, 3 articles lacked relevant data, 1 was identified as a conference abstract, leaving a final set of 18 articles (6–11, 14–25) (**Table 1**). The detailed literature screening process is shown in **Figure 1**.

**Table 1:** Basic characteristics of included literature.

First author	Year of publication	Author’s nationality	Study type	Subject source	Bipolar disorder diagnostic criteria	Number of samples of normal population	Unipolar disorder	Number of cases of bipolar disorder	The total	Number of cases of normal population in the training set	Number of cases of unipolar disorder in the training set	Number of cases of bipolar disorder in the training set	Total number of cases in the training set	Validation set generation method	Type of model used
Redlich, R.	2014	germany		Multicenter		58	58	58	174					Random sampling 10-fold cross-validation	SVM
Harry Rubin-Falcone, B.A.	2018	usa	Case control	Single-center	DSM-IV		26	26	52		26	26	52	Random sampling 10-fold cross-validation	SVM
Yantao Ma	2019	china	Control	Multicenter		228	255	360	843	228	255	360	843	10-fold cross-validation	RF,SVM,LASSO,LDA,LR
Haiteng Jiang	2020	china	Control	Single-center	DSM-IV	31	30	23	84	31	30	23	84	Random sampling 10-fold cross-validation	SVM
Yu, H.	2020	china		Case control			23	23						Random sampling 10-fold cross-validation	SVM
Sun, F.	2021	china	Case control	Single-center	DSM-IV		48	51	99					Random sampling 10-fold cross-validation	SVM
Jakub Tomasik	2021	UK	Case control	Website online recruitment	CIDI		126	187	313		126	187	313	5-fold cross-validation	XGBoost
Tao Yang	2021	china	Control	Single-center	DSM-IV	162	189	90	441	162	189	90	441	5-fold cross-validation	HYDRA
Sara Poletti	2021	Italy	Control		DSM-IV TR	32	127	81	240	32	127	81	240	Internal cross-validation	DL
Sawalha, J.	2021	Canada	Control	Single-center		53		74	127	53		74	127	5-fold cross-validation	SVM
						18		37	55	18		37	55	Random sampling 10-fold cross-validation	SVM
Jinkun Zeng	2023	China	Case control	Multicenter	ICD-10 criteria		918	242	1160		918	242	1160	Random sampling 10-fold cross-validation	LR SVM RF XGBoost
Zhao, Z. Y	2022	china	Case control	Single-center			44	26	70		44	26	70	5-fold cross-validation	
Tang, Q	2022	china	Control		DSM-IV TR	97	98	56	251	97	98	56	251	Random sampling 10-fold cross-validation	SVM
Margarette Sanchez, M	2022		Case control			0	71	71	142	0	71	71	142	5-fold cross-validation	XGBoost
Parker, G.	2022	Australia	Case control		DSM-III-R		29	161	190		29	161	190	Random sampling 10-fold cross-validation	Machine learning (ML)
Zhang, H.	2022					65	73	52						Random sampling 10-fold cross-validation	
Du, Y.	2022	china	Control	Single-center	ICD10 DSM-V	40		32	72	40		32	72	10-fold cross-validation	RF
Lu, F. M	2023		Control		DSM-IV-TR	97	58	95	250	97	58	95	250	Random sampling 10-fold cross-validation	SVM

### Study characteristics

Among the 18 primary studies included, the publication years ranged from 2014 to 2023, and they originated from 7 countries (Germany (17), USA (18, 25), China (6, 10, 11, 14–16, 19, 21–24), UK (20), Italy (8), Canada (9), Australia (7)), with sample sizes ranging from 52 to 1160. Among the included literature, 11 (6–9, 11, 14, 15, 18, 19, 21, 26) were single-center studies, while 6 (10, 16, 17, 20, 23, 25) were multi-center studies. All studies were case-control studies. 9 studies (6, 8–11, 14, 16, 17, 21) included healthy controls, while 16 studies (7, 8, 10, 11, 14–23, 25, 27) compared depression and bipolar disorder.

This study encompassed 18 studies, yielding a total of 28 constructed predictive models for bipolar disorder. Among these models, 1858 individuals had bipolar disorder, and the overall study population comprised 3152 individuals. There were 12 models utilizing depression as a control, 6 models using health as a control, 15 models incorporating neuroimaging as modeling variables, 9 models employing psychological assessment as modeling variables, and 4 models incorporating blood indicators as modeling variables. Various modeling methods were employed in the included studies, encompassing logistic regression, random forest, support vector machine, as well as other common machine learning (ML) methods (**Table 1**, **Supplementary Table S1**).

### Risk of bias in studies

**Figure 2:**
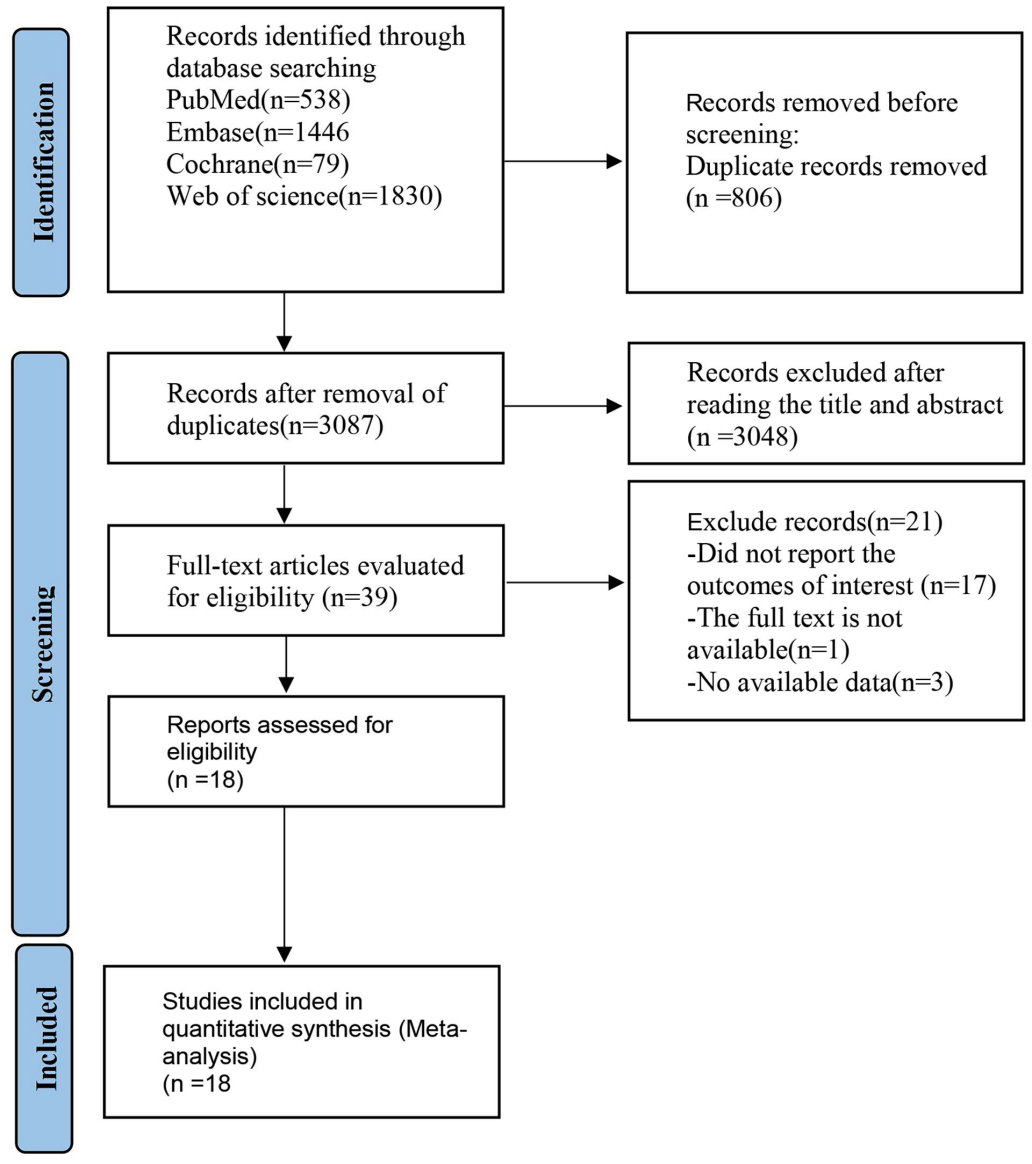
Assessment results of bias risk in included literature.

The included literature was evaluated for quality using QUADAS. Although all the included studies were case-control studies, our study was a review of machine learning. Among the included studies, 5 studies had bias in the evaluation of variables, which may affect the results of the models. Therefore, these 5 case-control studies had a high risk of bias in the domain of case selection. Although it was unclear whether the blinding method was implemented during the outcome assessment, considering the characteristics of machine learning, the risk of bias in the blinding of outcome assessors was low. In the original studies, the temporal relationship between the modeling variable evaluation and the outcome event was reasonable. In addition, all studies used gold standards for verification. Hence, all included studies had a low risk of bias in the implementation and interpretation of the reference standard. In addition, we believe that the evaluation of the clinical practicality of the included studies was reasonable, and thus the risk of bias was low. The evaluation results are shown in **Figure 2**.

### Meta analysis

#### Bipolar disorder vs. normal individuals

**Figure 3:**
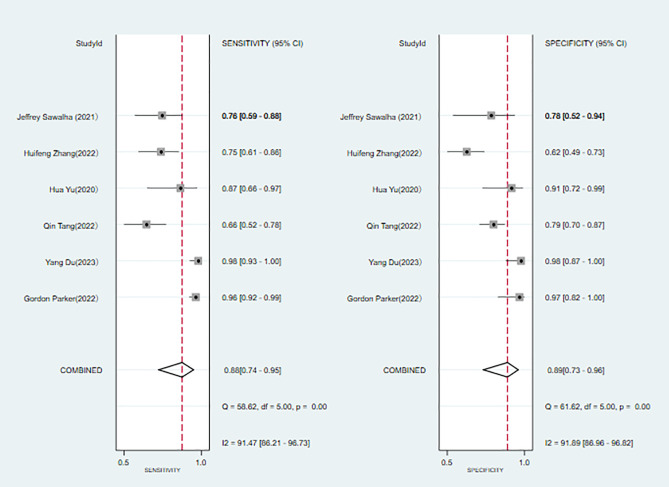
SROC of machine learning for discriminating bipolar disorder from healthy controls.

Heterogeneity assessment is a crucial step in determining the appropriateness of a precise estimate for combining data from various research sources. The SROC curve displayed a non-”shoulder-arm” distribution, indicating the absence of a threshold effect (**Figure 3**).

**Figure 4:**
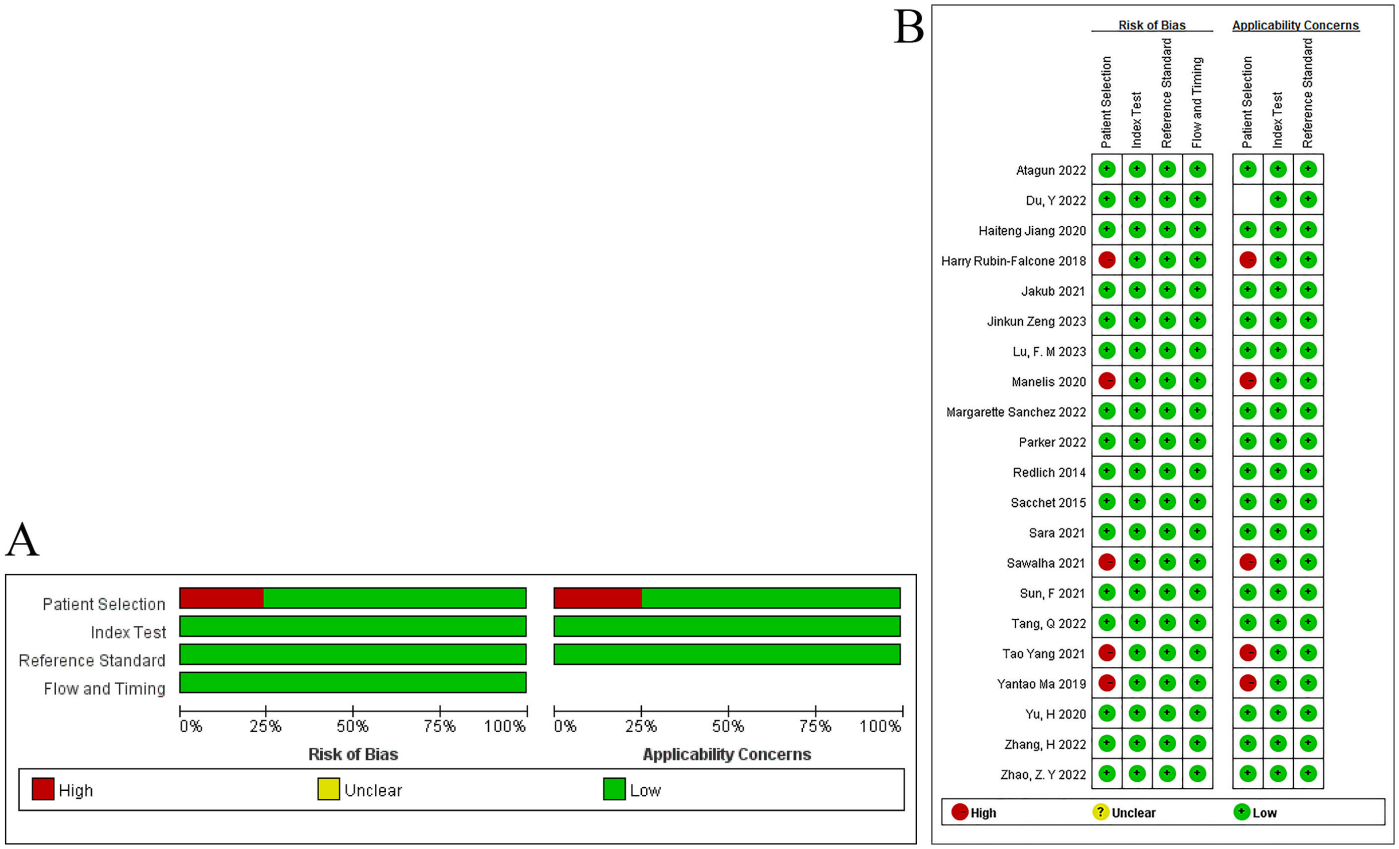
Forest plot of the sensitivity and specificity of machine learning for discriminating bipolar disorder from healthy controls.

A total of 6 (6, 7, 9–11, 22) studies were included in the analysis. The combined results predicted a sensitivity of 0.88 (95% CI: 0.740.95) for bipolar disorder, specificity of 0.89 (95% CI: 0.730.96), a positive likelihood ratio of 7.7 (95% CI: 2.721.9), a negative likelihood ratio of 0.14 (95% CI: 0.050.35), a diagnostic odds ratio of 57 (95% CI: 8385), and the area under the SROC curve (AUC) was 0.94 (0.920.96) (**Figure 4**).

**Figure 5:**
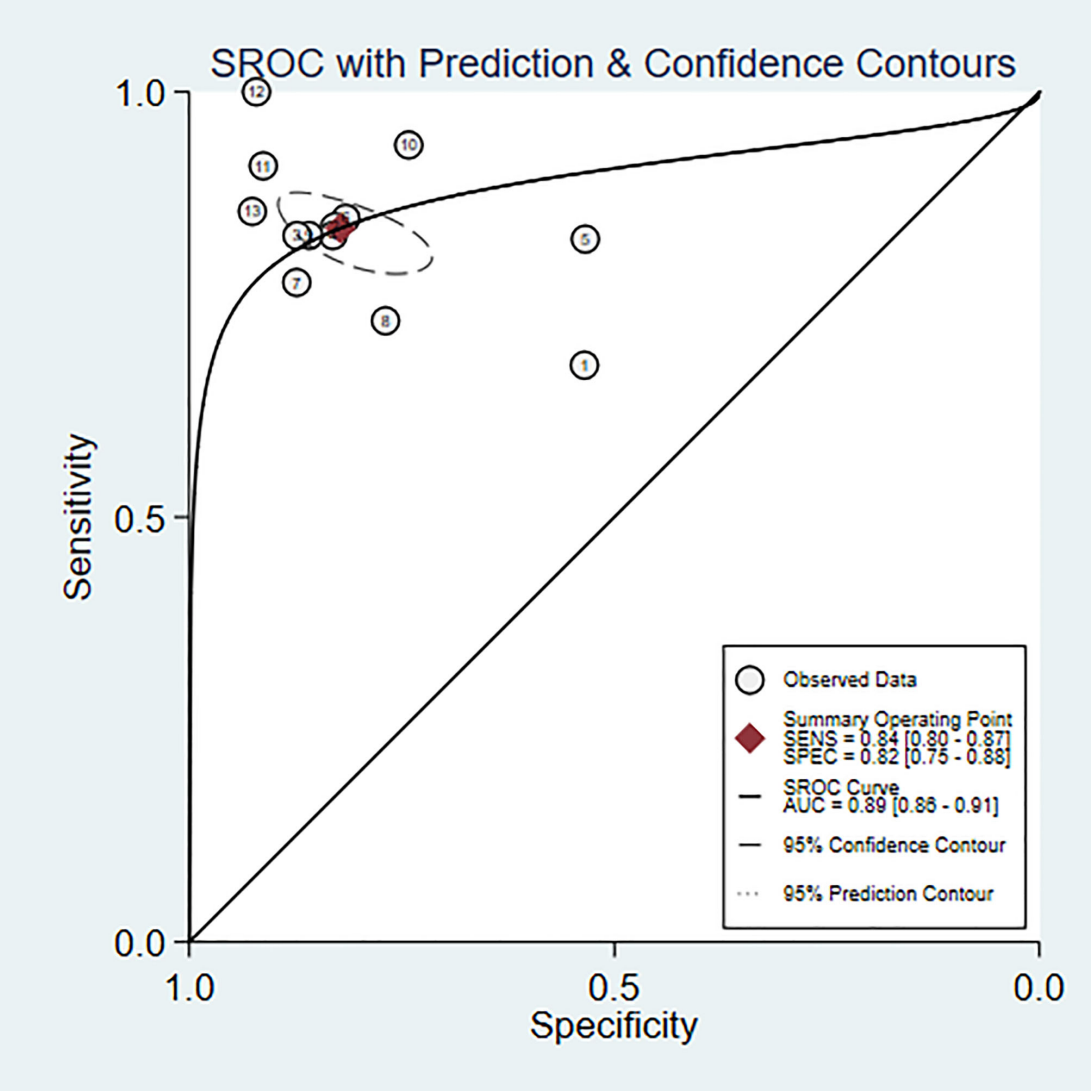
Deek’s funnel plot of machine learning for discriminating bipolar disorder from healthy controls.

**Figure 6:**
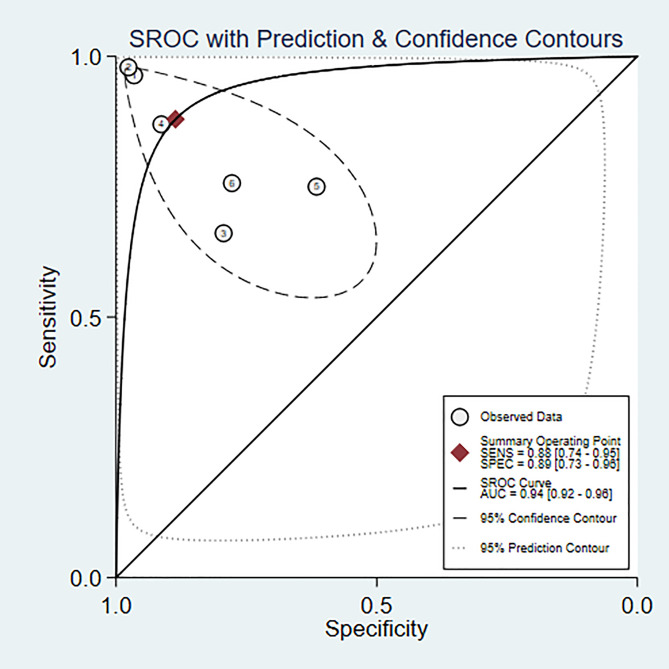
Nomogram of machine learning for discriminating bipolar disorder from healthy controls.

The models analyzed using Deek’s funnel plot displayed no evidence of publication bias (P=0.95) (**Figure 5**). Across the included studies, the prevalence of bipolar disorder was approximately 37% (**Figure 6**). Consequently, this prevalence was employed as the prior probability. In the realm of machine learning diagnosis for bipolar disorder, the actual probability of having the disorder is 0.74. In cases where machine learning diagnosis indicates non-bipolar disorder, the probability of actually having bipolar disorder is 0.1.

#### Bipolar disorder vs. depression

**Figure 7:**
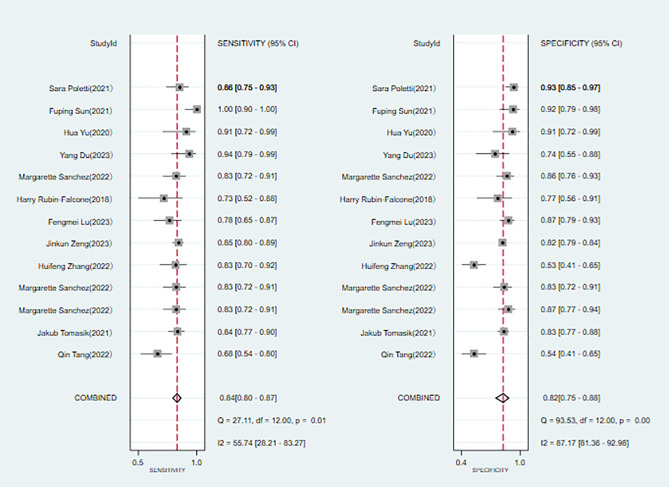
Deek’s funnel plot of machine learning for discriminating bipolar disorder from depression controls.

**Figure 8:**
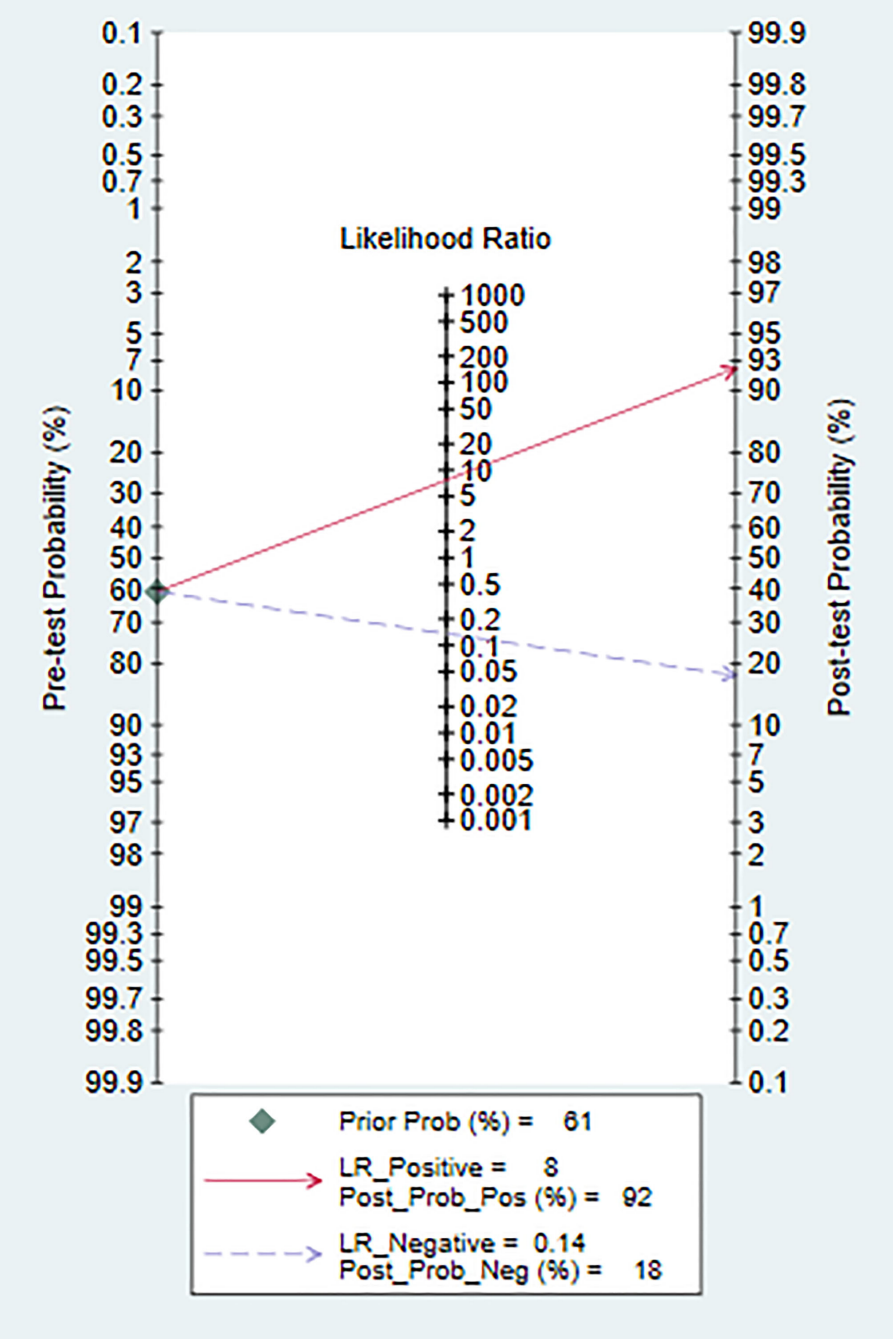
Nomogram of machine learning for discriminating bipolar disorder from depression controls.

Eleven studies (6, 8, 10, 11, 15, 18–20, 22, 23, 25) were included in the analysis. The combined results predict a sensitivity of 0.84 (95% CI: 0.80~0.87) and a specificity of 0.82 (95% CI: 0.75~0.88) for bipolar disorder. The positive likelihood ratio is 4.7 (95% CI: 3.2~6.9), the negative likelihood ratio is 0.20 (95% CI: 0.15~0.25), the diagnostic odds ratio is 24 (95% CI: 13~45), and the area under the SROC curve (AUC) is 0.89 (0.86~0.91) (**Figures 7**, **8**).

#### Subgroup analysis

Based on neuroimaging data as the basis for discrimination, a total of 10 studies (10, 11, 14, 15, 18, 19, 21, 22, 24, 25)were included. The combined results predict a sensitivity of 0.81 (95% CI: 0.76~0.86) and a specificity of 0.82 (95% CI: 0.75~0.87) for bipolar disorder, with a positive likelihood ratio of 4.4 (95% CI: 3.1~6.4) and a negative likelihood ratio of 0.23 (95% CI: 0.16~0.32), yielding a diagnostic odds ratio of 19 (95% CI: 10~37). The area under the SROC curve (AUC) is 0.88 (0.85~0.91) (**Supplementary Figures 1**, **2**).

Based on psychological assessment data (7, 9, 16, 17) as the basis for discrimination, a total of 5 studies were included. The combined results predict a sensitivity of 0.83 (95% CI: 0.74~0.90) and a specificity of 0.82 (95% CI: 0.73~0.88) for bipolar disorder, with a positive likelihood ratio of 4.6 (95% CI: 2.8~7.5) and a negative likelihood ratio of 0.20 (95% CI: 0.12~0.35). The diagnostic odds ratio is 23 (95% CI: 8~63), and the area under the SROC curve(AUC) is 0.90 (0.87~0.92) (**Supplementary Figures 3**, **4**).

Based on blood indicators as the basis for discrimination, a total of 3 studies (6, 8, 23)were included. The combined results predict a sensitivity of 0.91 (95% CI: 0.82~0.96) and a specificity of 0.90 (95% CI: 0.77~0.96) for bipolar disorder. The positive likelihood ratio is 8.9 (95% CI: 3.6~21.9), the negative likelihood ratio is 0.10 (95% CI: 0.05~0.22), and the diagnostic odds ratio is 88 (95% CI: 18~423). The area under the SROC curve (AUC) is 0.96 (0.94~0.97) (**Supplementary Figures 5**, **6**).

## Combined results

Based on the comprehensive analysis of all the studies, a total of 18·research papers were included. The combined results indicate a sensitivity of 0.84 (95% CI: 0.80~0.88) and a specificity of 0.83 (95% CI: 0.78~0.87) for diagnosing bipolar disorder. The positive likelihood ratio is 5.0 (95% CI: 3.7~6.7), the negative likelihood ratio is 0.19 (95% CI: 0.14~0.25), and the diagnostic odds ratio is 26 (95% CI: 15~46). The area under the SROC curve (AUC) is 0.90 (0.88~0.93) (**Supplementary Figures 7**, **8**).

## Discussion

### Summary of the main findings

We have determined that machine learning is a viable method for diagnosing bipolar disorder. In distinguishing bipolar disorder from healthy controls, the sensitivity is 0.88, and the specificity is 0.89. When discriminating between bipolar disorder and depression, the sensitivity is 0.84, and the specificity is 0.82. The primary modeling variables are derived from neuroimaging, blood indicators, and psychological assessments. Notably, the model constructed from neuroimaging demonstrates relatively ideal sensitivity and specificity.

Various neuroimaging techniques, including resting-state MRI, functional MRI, electroencephalogram, resting-state electroencephalogram, and gray matter volume, are the main diagnostic tools for neuroimaging modeling. A study by (22) highlights the significance of resting-state MRI, achieving an accuracy of 91.3%, although results vary, with some studies (17)reporting only 69%. Additionally, a study (21) suggests that the brain structure of patients with bipolar disorder may not be entirely specific. However, the diagnostic value (15) of functional MRI is relatively higher.

### Comparison with previous studies (other reviews)

We observed that other researchers have explored non-invasive discrimination methods for bipolar disorder, primarily relying on imaging methods. In comparison with other machine learning predictive models, Hao Li et al (28) utilized magnetic resonance imaging to predict bipolar disorder based on differences in gray matter volume and ReHo values. The accuracy is 0.875 (95% CI: 0.7250.953), sensitivity is 0.864 (95% CI: 0.640.964), and specificity is 0.889 (95% CI: 0.639~0.98). In a review by some researchers (29) on applying machine learning to diagnose mental disorders, they found that the accuracy of using MRI structural imaging can reach 100%, functional MRI imaging can achieve 98.7% accuracy, and multimodal accuracy can reach 99.5% (30–32). Although their sensitivity is high, the specificity is insufficient (33), and the sample size is small (28).

In our research, we have identified that modeling variables for the identification of bipolar disorder using AI are crucial. In the studies we have incorporated, the modeling variables primarily derive from neuroimaging and blood indicators. We have also found that, based on psychological assessment, there is no particular advantage, as their sensitivity and specificity are quite similar. Blood indicators demonstrate optimal sensitivity and specificity, and in subsequent studies, we may consider incorporating blood indicators into the differential diagnosis of bipolar disorder. However, it may be necessary to include more blood indicator data, such as inflammatory factors (32). Some studies (23)suggest that blood indicators are the most effective diagnostic tool. Different studies encompass different variables when blood indicators are used as research variables. Zeng, Jinkun et al (23)included 27 indicators of complete blood cell counts and 17 indicators of blood biochemical markers. Poletti, S et al (8) included 54 blood indicators, while Du, Y et al (6)included 15 blood metabolites. In subsequent studies, we need to further screen for risk factors and identify the blood indicators that need to be distinguished.

In addition to focusing on modeling variables, the selection of a model often involves two challenging decisions and a balance between two aspects: choosing between interpretability and accuracy of the model. Models with good interpretability (such as decision trees, logistic regression, and COVS regression) demonstrate strong diagnostic capabilities and predictive performance. However, concerns exist about their interpretability. For example, in the application of neural networks and deep learning to the medical field, it is challenging to avoid models with poor interpretability, especially in machine learning models for imaging. This represents a challenge that needs addressing in future research.

### Advantages

Compared to prior studies utilizing big data for bipolar disorder diagnosis, our study demonstrates an overall higher accuracy, surpassing that of previous research (82% accuracy) (34). Furthermore, we included a large number of variables, such as neuroimaging data, blood indicators, and psychological assessment results, rather than just incorporating one-sided data. Additionally, in the studies included, various machine learning methods were incorporated into research. A study (29) suggests that while big data plays an important role, different machine learning methods also significantly contribute to diagnosis. The presentation of all results is based on machine learning. The main machine learning algorithms include deep learning (DL), support vector machine (SVM), random forest (RF), k-nearest neighbors (KNN), logistic regression, gradient boosting, and decision tree. In terms of machine learning applied to disease diagnosis, some (29) argue that the logistic regression algorithm has the highest accuracy of 0.85, but in practice, SVM is used the most (35).

### Limitations

There are still some deficiencies in our study. First, although we conducted a systematic literature search, there is still a lack of literature on using blood as a detection indicator, with only 4 studies included. The limited number of included literature may impact the research results. Second, we believe that discussing Type I and Type II bipolar disorder is quite necessary. However, upon reviewing the included studies again, we found that these studies largely failed to clearly differentiate between Type I and Type II bipolar disorder. Therefore, our study was unable to proceed with a more in-depth discussion. Third, the inconsistent diagnostic criteria may also cause some variances. The psychological assessment instruments are insufficiently broad, and there is no evaluation of disease protective variables. Fourth, the fact that positive findings are more likely to be published could lead to potential publication bias, which should be taken into account when applying the results. Fifth, the literature search is limited to English only, which may result in bias towards certain languages.

## Conclusions

Machine learning has a certain predictive value for the diagnosis of bipolar disorder, with SVM being the most widely used method. However, there is a need to further discriminate the specific indicators included and make the research indicators more specific to achieve a higher level of accuracy and provide a solid basis for clinical diagnosis.

## Data Availability

The original contributions presented in the study are included in the article/**Supplementary Material**. Further inquiries can be directed to the corresponding authors.
