# Supplementary material for: Machine learning for the diagnosis accuracy of bipolar disorder: a systematic review and meta-analysis
Source: Front Psychiatry. 2025 Jan 28;15:1515549. doi: 10.3389/fpsyt.2024.1515549 (PMC11810903; doi:10.3389/fpsyt.2024.1515549)
Supplement: Supplementary file 1 [file SupplementaryFile1.docx]

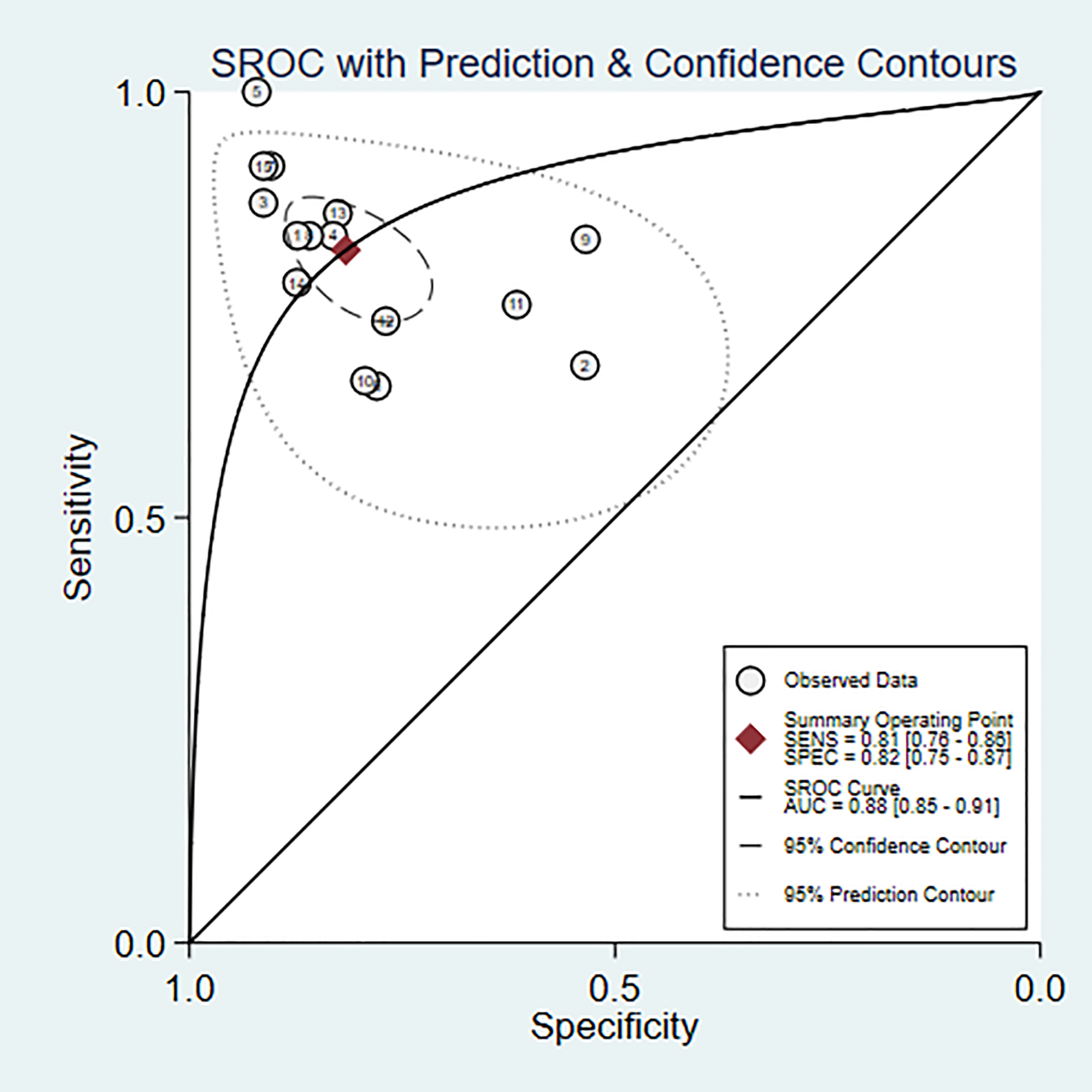


**Figure S1:** SROC of neuroimaging for predicting bipolar disorder.


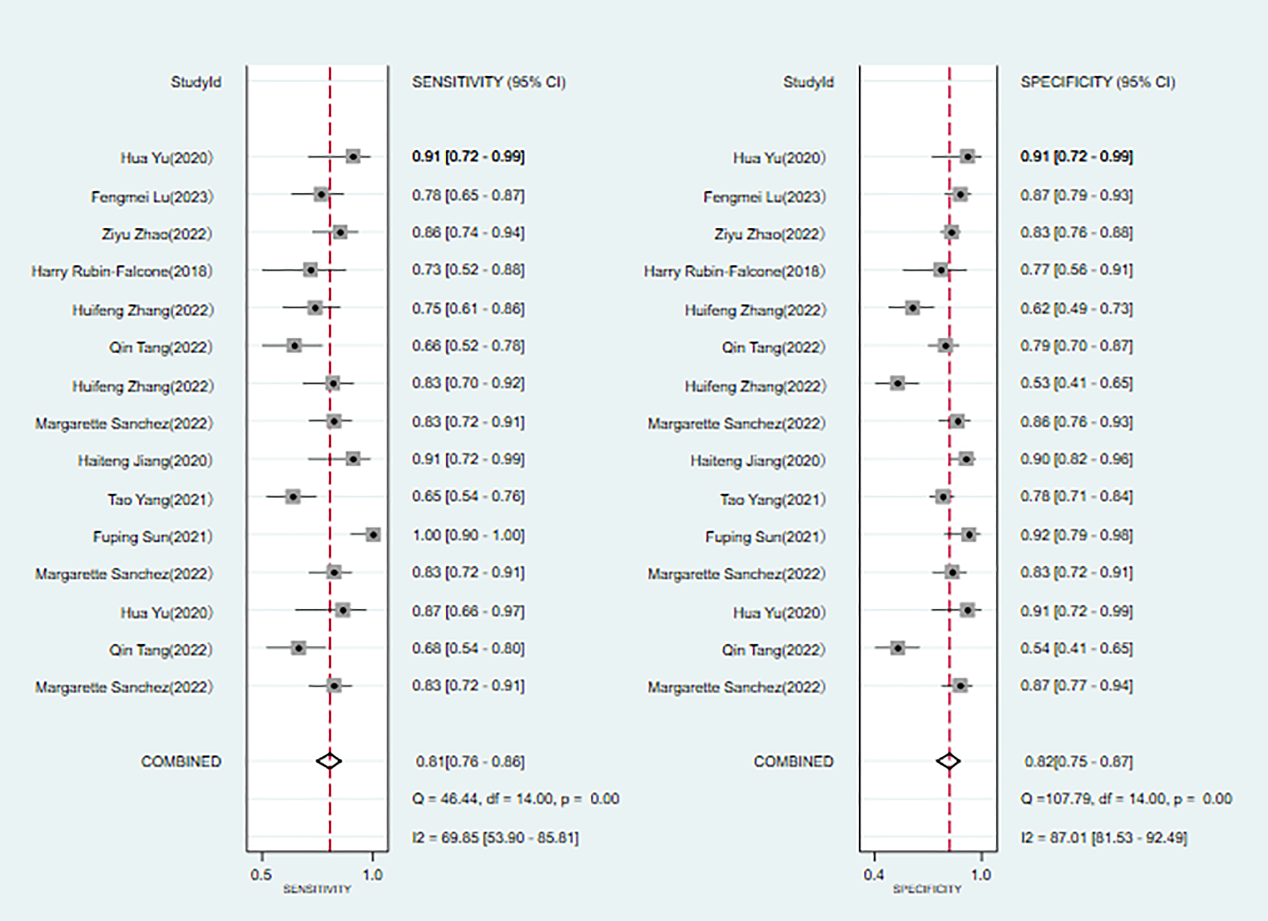


**Figure S2:** Forest plot of sensitivity and specificity of neuroimaging for predicting bipolar disorder.


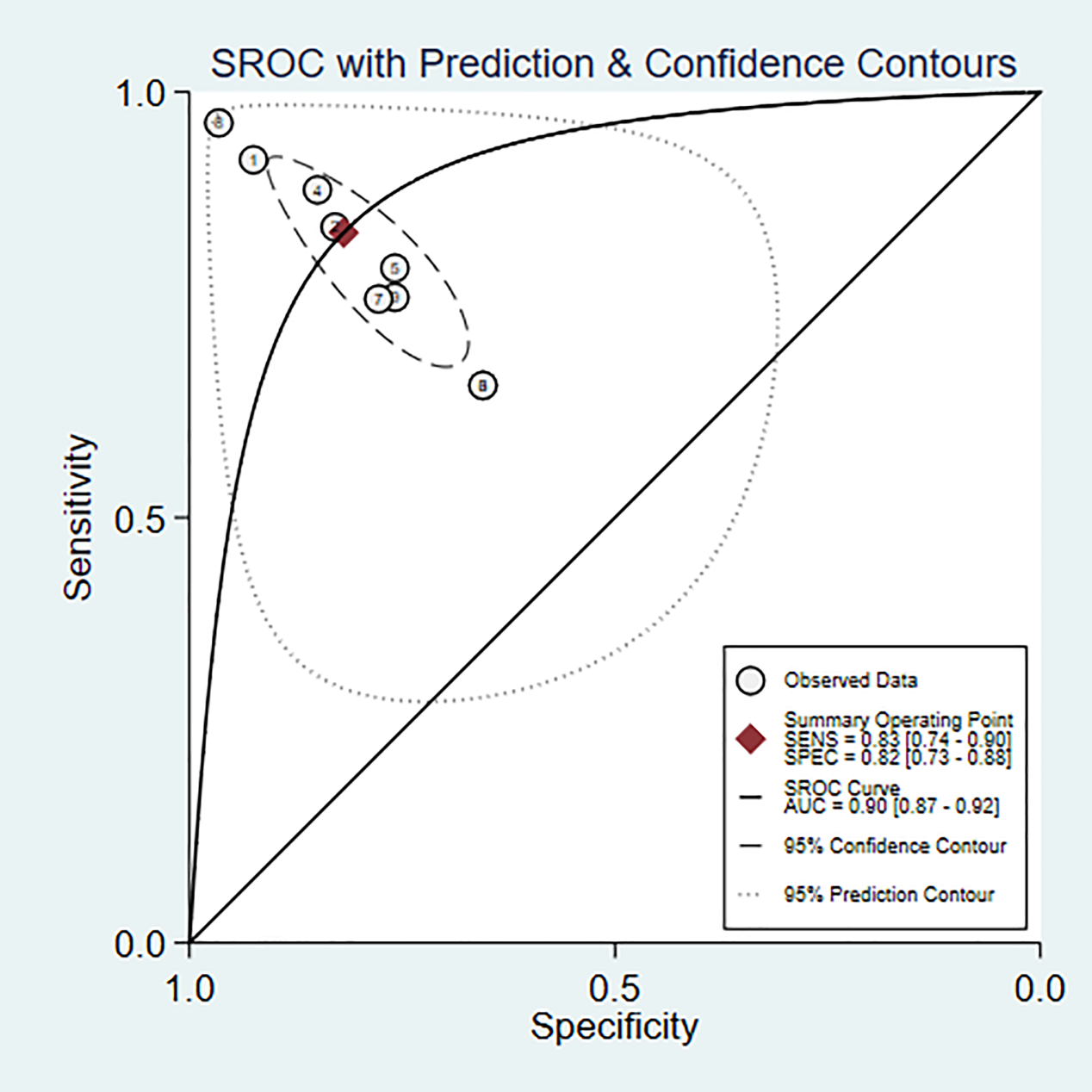


**Figure S3:** SROC of psychological assessments for predicting bipolar disorder.


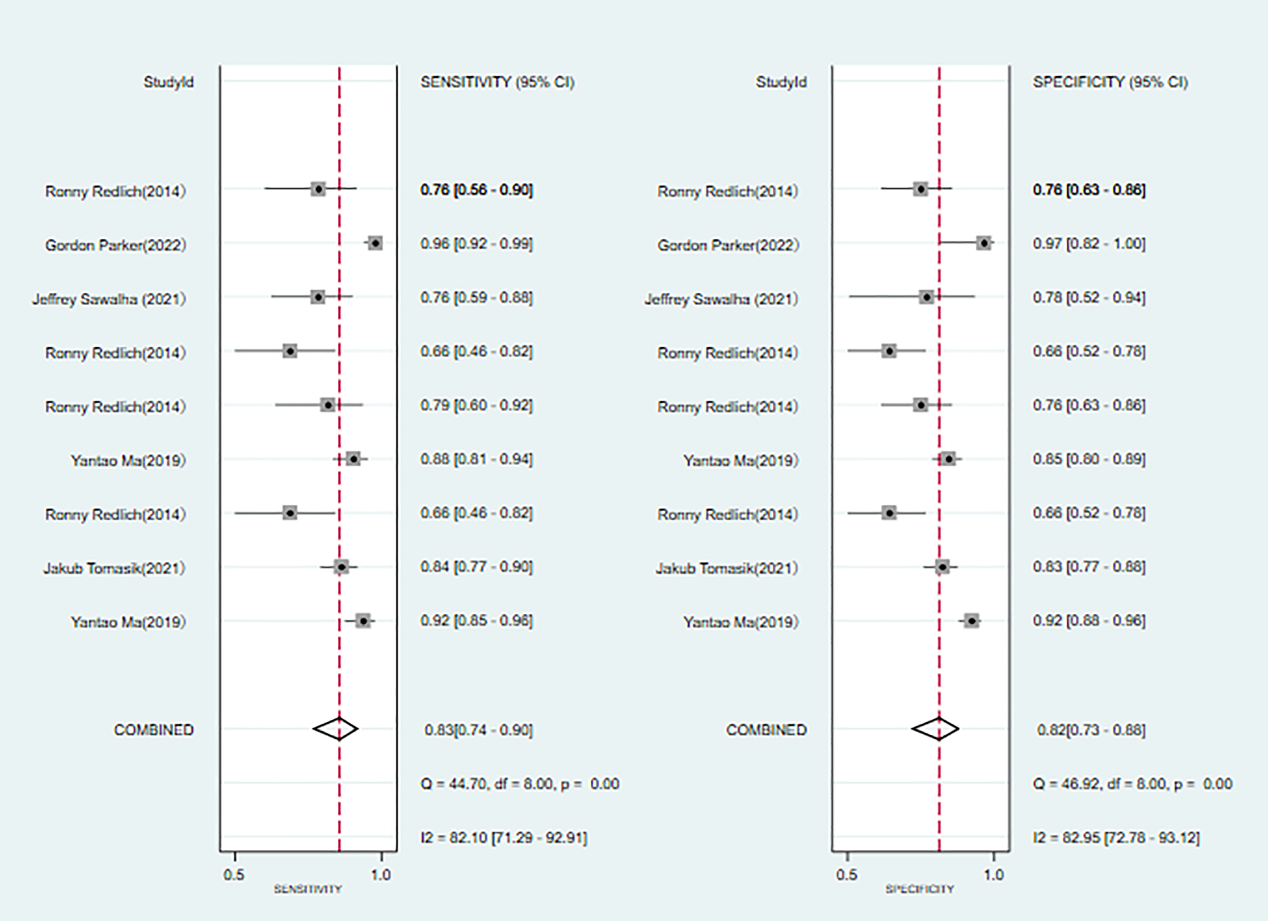


**Figure S4**: Forest plot of sensitivity and specificity of psychological assessments for predicting bipolar disorder.


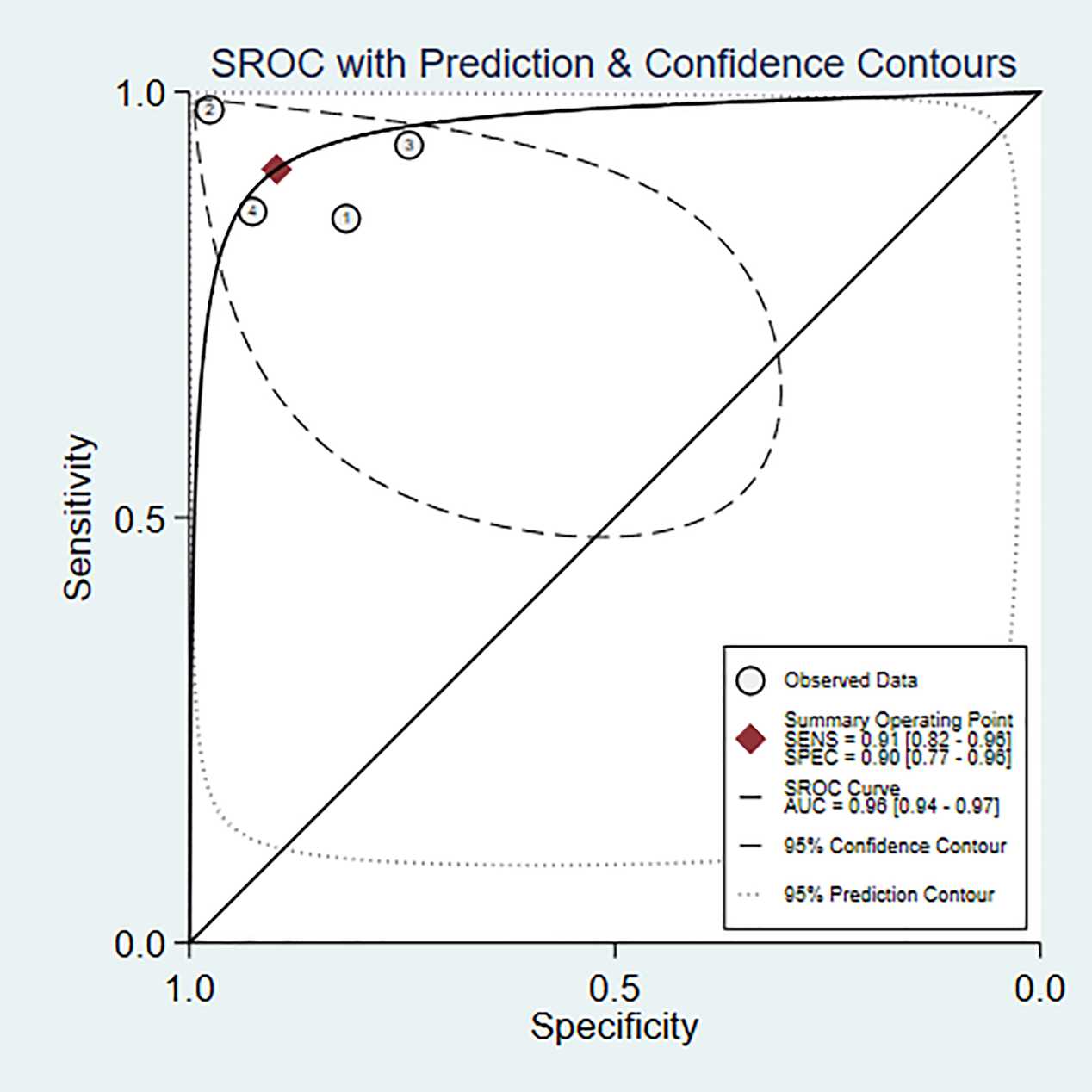


**Figure S5:** SROC of blood indicators for predicting bipolar disorder.


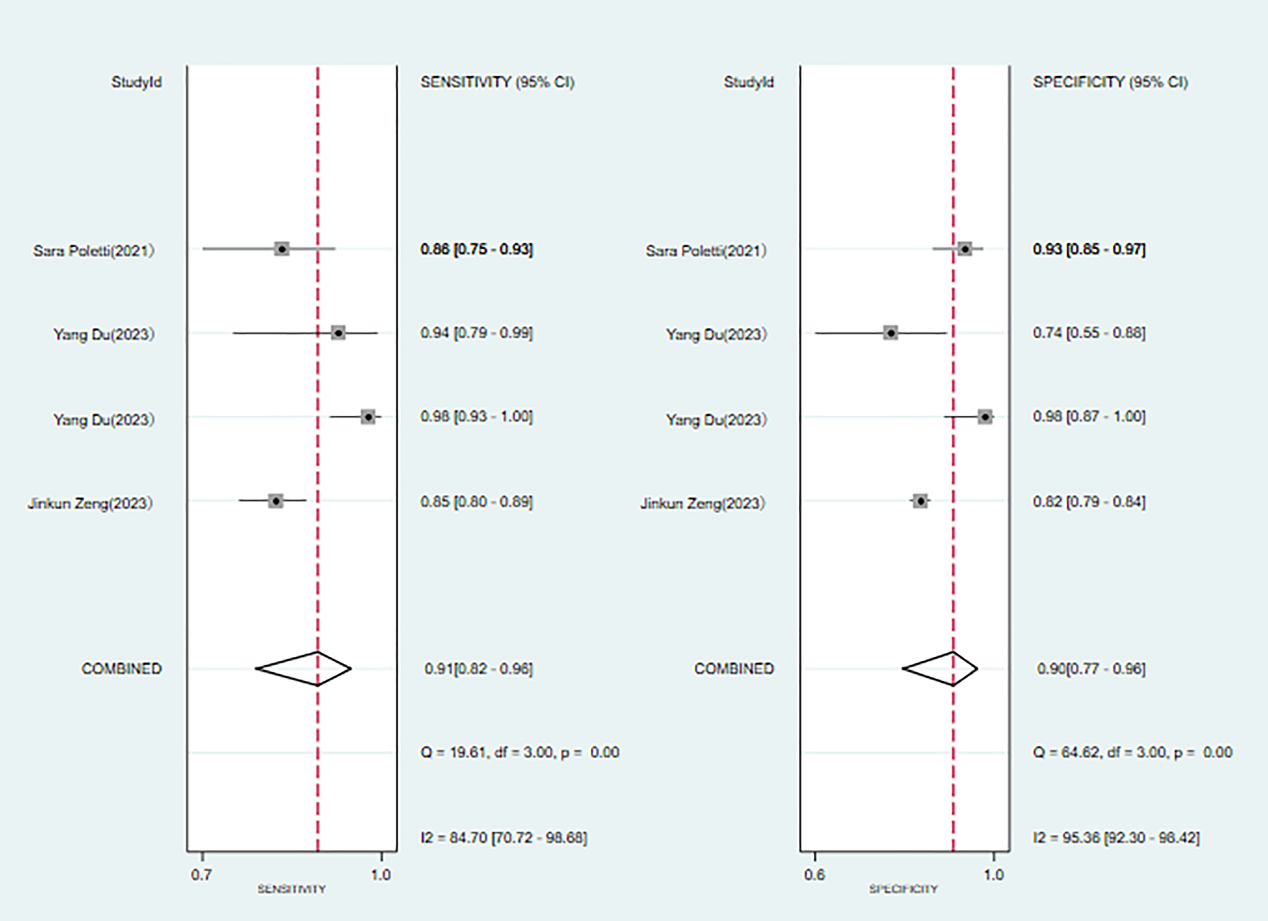


**Figure S6:** Forest plot of sensitivity and specificity of blood indicators for predicting bipolar disorder.


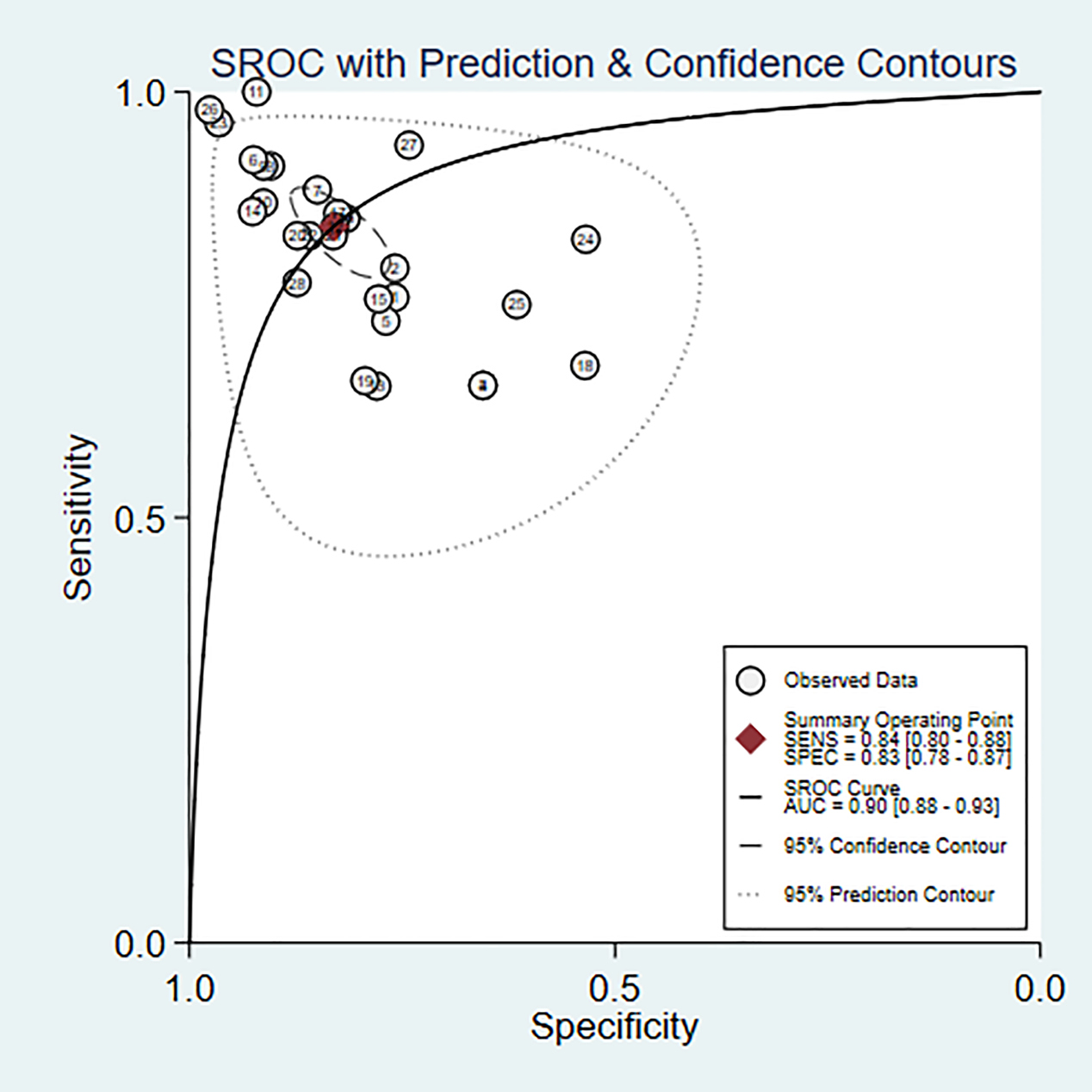


**Figure S7:** SROC of all data for predicting bipolar disorder.


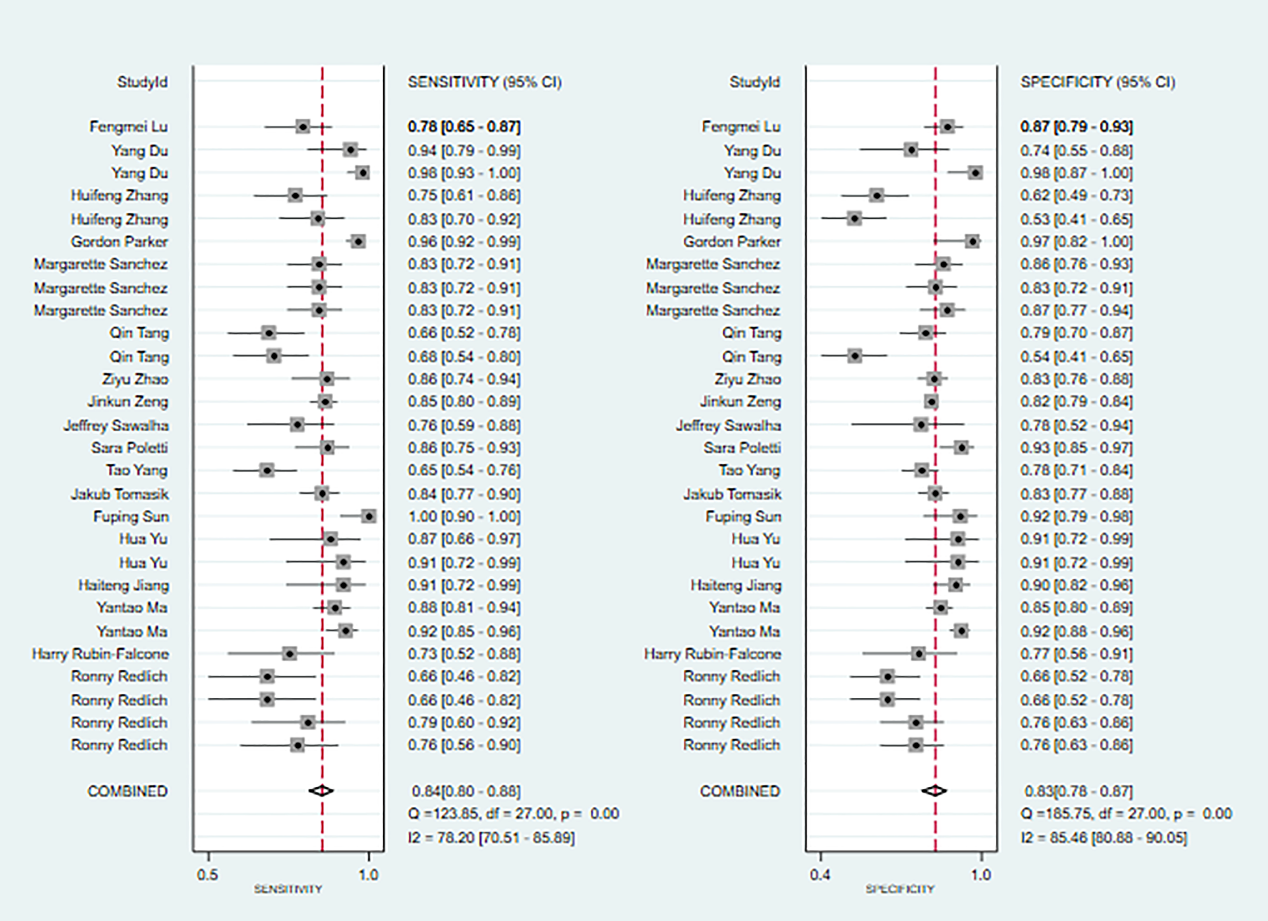


**Figure S8**: Forest plot of sensitivity and specificity of all data for predicting bipolar disorder.
