# Supplementary material for: Machine learning for the diagnosis accuracy of bipolar disorder: a systematic review and meta-analysis
Source: Front Psychiatry. 2025 Jan 28;15:1515549. doi: 10.3389/fpsyt.2024.1515549 (PMC11810903; doi:10.3389/fpsyt.2024.1515549)
Supplement: Supplementary file 2 [file Table1.docx]

Table S1 Summary of variablesof the included models

| No. | First author | Year of publication | Modeling variables |
| --- | --- | --- | --- |
| 1 | Redlich, R. | 2014 | Magnetic resonance imaging |
| 2 | Harry Rubin-Falcone, B.A. | 2018 | Magnetic Resonance Imaging |
| 3 | Yantao Ma | 2019 | Bipolar Diagnosis Checklist in Chinese (BDCC) |
| 4 | Haiteng Jiang | 2020 | Resting-state magnetoencephalography (MEG) |
| 5 | Yu, H. | 2020 | Whole brain resting-state functional connectivity (rs-FC) |
| 6 | Sun, F. | 2021 | Resting-state functional magnetic resonance |
| 7 | Jakub Tomasik | 2021 | Elevated mood, grandiosity, talkativeness, recklessness and risky behaviour |
| 8 | Tao Yang | 2021 | Brain structural data |
| 9 | Sara Poletti | 2021 | Plasma concentrations of the following immune analytes were determined using the bead-based Luminex system based on xMAP technology (Bio-Rad Laboratory, Hercules, CA, USA): Cytokines: IL1β, IL-1rα, IL-2, IL-4, IL-5, IL-6, IL-7, IL-8, IL-9, IL-10, IL-12, IL-13, IL-15, IL-16, IL-17,IFNγ, TNFα; Macrophage migration inhibitory factor (MIF); Chemokines: C-C motif ligand 1 (CCL1), CCL2, CCL3, CCL4, CCL5,CCL8, CCL7, CCL11, CCL13, CCL15, CCL17, CCL19, CCL20, CCL21, CCL22, CCL23, CCL24, CCL25, CCL26, CCL27; C-X-C motif chemokine (CXCL)1, CXCL2, CXCL5,CXCL6, CXCL8, CXCL9, CXCL10, CXCL11, CXCL12, CXCL13,CXCL16, CX3CL1; Growth factors: fibroblast growth factor basic (bFGF), Granulocyte Colony Stimulating Factor (G-CSF), Granulocyte Macrophage Colony Stimulating Factor (GM-CSF), Platelet-Derived Growth Factor Beta (PDGF-B), Vascular Endothelial Growth Factor (VEGF). Assays were performed on Luminex 200 system. Samples were analyzed according to manufacturer’s instructions |
| 10 | Sawalha, J. | 2021 | The Cambridge Neuropsychological Test Automated Battery（CANTAB） |
| 11 | Jinkun Zeng | 2023 | (1) clinical features of disease duration and age of onset, (2) biochemical markers of albumin, low density lipoprotein (LDL), and potassium, and (3) complete blood count-derived biomarkers of white blood cell count (WBC), platelet-to-lymphocyte ratio (PLR), and monocytes (MONO) |
| 12 | Zhao, Z. Y | 2022 | Electroencephalography (EEG) |
| 13 | Tang, Q | 2022 | The resting-state functional magnetic resonance |
| 14 | Margarette Sanchez, M | 2022 | Electroencephalography (EEG) |
| 15 | Parker, G. | 2022 | Assessing 96 putative manic/hypomanic symptoms scale |
| 16 | Zhang, H. | 2022 | The whole-brain "high-order functional connectivity (HOFC) |
| 17 | Du, Y. | 2022 | The 15 exosomal metabolites |
| 18 | Lu, F. M | 2023 | The dynamic FC (dFC) |
